# A Modulation Method for Tunnel Magnetoresistance Current Sensors Noise Suppression

**DOI:** 10.3390/mi15030360

**Published:** 2024-03-01

**Authors:** Shuaipeng Wang, Haichao Huang, Ying Yang, Yanning Chen, Zhen Fu, Zhenhu Jin, Zhenyu Shi, Xingyin Xiong, Xudong Zou, Jiamin Chen

**Affiliations:** 1 Beijing Smart-Chip Microelectronics Technology Co., Ltd., Beijing 102299, China; wangshuaipeng@sgchip.sgcc.com.cn (S.W.);; 2 State Key Laboratory of Transducer Technology, Aerospace Information Research Institute, Chinese Academy of Sciences, Beijing 100190, China

**Keywords:** TMR current sensor, modulation and demodulation, noise suppression

## Abstract

To mitigate the impact of low-frequency noise from the tunnel magnetoresistance (TMR) current sensor and ambient stray magnetic fields on weak current detection accuracy, we propose a high-resolution modulation-demodulation test method. This method modulates and demodulates the measurement signal, shifting low-frequency noise to the high-frequency band for effective filtering, thereby isolating the target signal from the noise. In this study, we developed a Simulink model for the TMR current sensor modulation-demodulation test method. Practical time-domain and frequency-domain tests of the developed high-resolution modulation-demodulation method revealed that the TMR current sensor exhibits a nonlinearity as low as 0.045%, an enhanced signal-to-noise ratio (SNR) of 77 dB, and a heightened resolution of 100 nA. The findings indicate that this modulation-demodulation test method effectively reduces the impact of low-frequency noise on TMR current sensors and can be extended to other types of resistive devices.

## 1. Introduction

TMR sensors, with their high sensitivity, linearity, response frequency, and extensive linear range, are regarded as highly promising for current measurement in power systems, offering picotesla (pT)-level resolution and frequency detection from DC to MHz. However, the prevalent 1/f noise within the low-frequency region significantly impairs the magnetic field detection capabilities of TMR sensors. To enhance magnetic field signal quality, modulation of TMR sensors’ output signals is essential. Presently, interface circuits predominantly employ auto-zeroing and chopping techniques to mitigate noise and misalignment in the low-frequency voltage output of TMR sensors [1]. Owing to aliasing issues, the auto-zeroing technique not only falls short in fully eliminating the 1/f noise from the output signal but also intensifies low-frequency thermal noise. Chopping techniques transfer noise to a different frequency band than the signal, effectively achieving signal-to-noise separation. A capacitor-coupled chopping amplifier can effectively suppress noise, yet this approach may reduce the input impedance of the instrumentation amplifier to a certain degree [2,3,4,5,6]. Current feedback structures can fundamentally address this issue, with recent research focusing on chopping amplifiers based on these structures [7,8,9,10]. Within chopping technology, designing low-frequency low-pass filters presents increased complexity in CMOS circuits, leading to the rising trend of employing high-frequency ripple suppression loops as alternative solutions [11]. Regarding modulation-demodulation, the ΔΣ modulator structure is extensively utilized in digital chips and integrated into TMR magnetic sensor interface circuits [12,13]. Beyond circuit structure adjustments for 1/f noise, employing dual-resonator synchronous modulation based on MEMS structures to transition the magnetic field from low to high frequency also proves effective in eliminating 1/f noise [14,15].

This study introduces a high-resolution modulation-demodulation test method designed to reduce the impact of low-frequency noise during testing and enhance the accuracy of weak current detection in TMR current sensors. The method entails modulating and demodulating the TMR sensor’s output signal, thereby shifting low-frequency noise to the high-frequency band, filtering it out with a low-pass filter, and consequently isolating the target signal from the noise. The core contributions of this paper involve establishing the proposed high-resolution modulation-demodulation test method for TMR current sensors and implementing practical time-domain and frequency-domain experiments to validate the method’s effectiveness in noise reduction.

## 2. Principle

**Figure 1:**
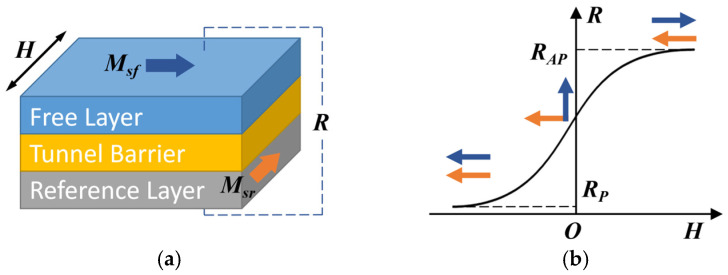
Schematic diagram of MTJs: (**a**) MTJs film layer structure; (**b**) Curve of MTJs resistance with magnetic field.

TMR typically consists of serially connected magnetic tunnel junctions (MTJs). Its structure is schematically shown in Figure 1a, with the reference layer, the tunnel layer, and the free layer from bottom to top. $M_{sf}$ and $M_{sr}$ respectively represent the magnetization directions of the free layer and reference layer, *H* represents the applied external magnetic field, and *R* represents the tunnel magnetoresistance value. Under the influence of an external magnetic field, the magnetization direction of the reference layer remains fixed, while the magnetization direction of the free layer changes with the variation of the external magnetic field. The magnitude of the tunnel magnetoresistance of the MTJ depends on the relative orientation of the magnetization directions of the pinned layer and the free layer, and its R-H curve is shown schematically in Figure 1b. When the magnetization directions of the free layer and the reference layer are parallel, the MTJ presents a low resistance state; when the applied magnetic field is zero, the magnetization directions of the free layer and the reference layer are orthogonal, and the MTJ presents a zero-resistance state. When the magnetization of the free layer and the reference layer are antiparallel, the MTJ presents a high-resistance state. Within a certain range of magnetic fields, the MTJ resistance value is proportional to the strength of the applied magnetic field.

**Figure 2:**
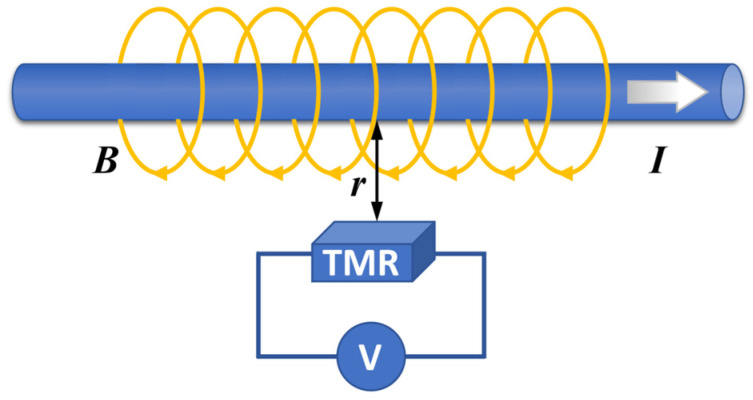
Schematic diagram of current measurement by TMR sensor.

The principle of the TMR current sensor is shown in Figure 2. When a current *I* is passed through the conductor of a power system, a spiral magnetic field is generated around the conductor. According to Biot–Savart’s law, the magnetic flux density *B* is proportional to the current *I*. By placing the TMR sensor around the conductor, the value of the current is deduced from the magnetic field generated by the current sensed by the TMR sensor. When the sensitive direction of the TMR sensor is parallel to the direction of the magnetic field generated by the current, changing the value of the current, the magnetic field around the conductor, and the value of the magnetoresistance inside the TMR sensor also change accordingly. As the TMR sensor internally uses a Wheatstone bridge structure, the change in the magnetic field is converted into a linear relationship with its differential output voltage; the output voltage indirectly reflects the value of the current through the conductor.

**Figure 3:**
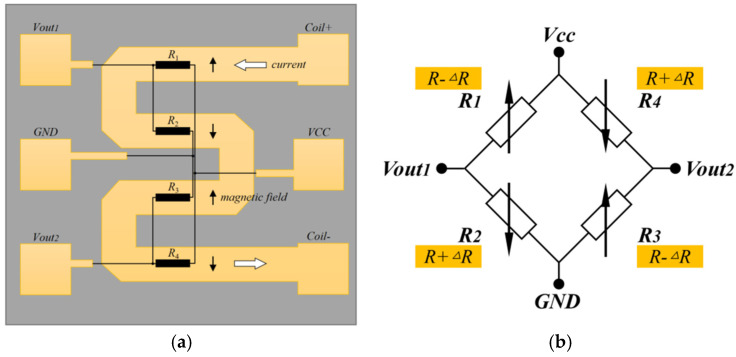
TMR current sensor with an integrated coil inside the chip: (**a**) Structure of TMR current sensors with internally integrated coils; (**b**) TMR Wheatstone full bridge structure.

In some of these sensors, the sensitivity of the TMR current sensor can be changed by adjusting the distance *r* between the conductor and the TMR sensing element, which in turn leads to different measurement ranges. In order to reduce the influence of stray magnetic fields when measuring weak currents of microamps and to enhance the signal-to-noise ratio of the measurement signal, the distance between the conductors and the TMR sensing element can be minimized by integrating the conductors inside the chip using the MEMS technology, as shown in Figure 3a. R1–R4 are four identical TMR resistor strips with the same direction of the magnetization of their pinned layers. The internal conductors create meandering current paths such that the current flow direction within R1 and R3 is right-to-left, and the current flow direction within R2 and R4 is left-to-right. The current generates a magnetic field of the same size and opposite direction around the two sets of resistors, with the magnetic field parallel to the magnetization direction of the pinned layers of R1 and R3 and antiparallel to the magnetization direction of the pinned layers of R2 and R4. At this time, the magnetic field causes the magnetization direction of the free layer, which is originally perpendicular to the magnetic field, to be deflected, resulting in a decrease in the resistance of R1 and R3 and an increase in the resistance of R2 and R4. The full bridge structure consisting of four TMR sensing elements with the same sensitivity direction is shown in Figure 3b, which realizes a differential output with the output voltage being the difference between Vout1 and Vout2.

## 3. Simulation

### 3.1. Analysis of TMR Current Sensor Modulating Test Method

**Figure 4:**
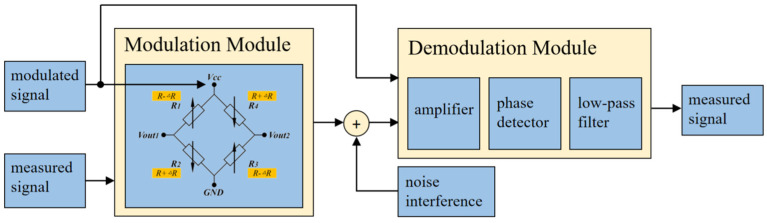
Block diagram of the modulation-demodulation test method for the TMR current sensor.

In current detection systems, the physical characteristics of TMR sensors and the testing system impose limitations, resulting in severe noise contamination and some extent of distortion in addition to the measurement signal output by the sensor. When detecting weak currents, the output signal of the sensor is generally very weak, making it crucial to separate the target signal from the noise-contaminated output signal. Among the noises, low-frequency 1/f noise has a noticeable impact on the accuracy of current detection. Therefore, it is necessary to eliminate this noise as much as possible to improve the resolution of current detection. Considering the characteristics of TMR current sensors, a high-resolution modulation-demodulation testing method is proposed in this paper. This method modulates the frequency of the low-frequency noise signal in the output signal to the high-frequency band and then demodulates it to recover the target signal. Finally, the high-frequency band is filtered out using a low-pass filter, resulting in a signal that only contains the components of the target signal. Figure 4 illustrates the schematic diagram of the modulation-demodulation method, which consists of a modulation module and a demodulation module.

The output voltage formula for the TMR Wheatstone full bridge is:

(1) $$V_{out}=\left(V_{out1}-V_{out2}\right)+V_{n}=V_{CC}\times \frac{\Delta R}{R}+V_{n}$$

where $V_{out}$ is the full bridge output signal; $V_{out1}$ and $V_{out2}$ are the signals from the differential ports; $V_{n}$ is the noise; $V_{CC}$ is the supply voltage; Δ*R* is the amount of change in a single TMR resistor; and *R* is the TMR zero-field resistance.

In the modulation module, the integrated coil in the TMR current sensor is energized with the low-frequency AC current $I\cos\omega_{o}t$. In addition to the DC voltage power supply, a high-frequency, small-amplitude AC voltage $V_{AC}\cos\omega_{g}t$ is added to the supply voltage as a modulating signal so that the output signal of the TMR full bridge is:

(2) $$V_{out}\left(t\right)=\left(V_{DC}+V_{AC}\cos\omega_{g}t\right)\times A\cos\omega_{o}t+V_{n}\left(t\right)$$

where $V_{DC}$ is the amplitude of the DC supply voltage; $V_{AC}$ is the amplitude of the AC supply voltage; $\omega_{g}$ is the frequency of the AC supply voltage; *A* is the TMR current sensor sensitivity; and $\omega_{o}$ is the frequency of the AC current to be measured.

Therefore, the process of TMR current sensor output can be regarded as a modulation process of the signal. Simplifying Formula (2), set the measurement signal as $S\left(t\right)=\alpha_{1}\cos\omega_{1}t$, the modulating signal as $g\left(t\right)=\alpha_{2}\cos\omega_{2}t$, the modulating signal adding bias as *β*, the low-frequency noise as $n\left(t\right)$, and the output signal of the full bridge as $F\left(t\right)$. The output of the TMR current sensor can be expressed as:

(3) $$\begin{array}{ll} F\left(t\right) & =S\left(t\right)\times g\left(t\right)+n\left(t\right)\\ & =\alpha_{1}\cos\omega_{1}t\times \left(\alpha_{2}\cos\omega_{2}t+\beta \right)+n\left(t\right)\\ & =\frac{\alpha_{1}\alpha_{2}}{2}\left[\cos\left(\omega_{1}+\omega_{2}\right)t+\cos\left(\omega_{2}-\omega_{1}\right)t\right]+\alpha_{1}\beta \cos\omega_{1}t+n\left(t\right) \end{array}$$

Set the demodulation signal as $F_{d}\left(t\right)$, and the signal demodulation uses the same signal $g\left(t\right)=\alpha_{2}\cos\omega_{2}t$ in the AC part of the modulating signal, then the time domain expression of the demodulation signal $F_{d}\left(t\right)$ is:

(4) $$\begin{array}{ll} F_{d}\left(t\right) & =F\left(t\right)\times g\left(t\right)\\ & =\left\{\frac{\alpha_{1}\alpha_{2}}{2}\left[\cos\left(\omega_{1}+\omega_{2}\right)t+\cos\left(\omega_{2}-\omega_{1}\right)t\right]+\alpha_{1}\beta \cos\omega_{1}t+n\left(t\right)\right\}\times \alpha_{2}\cos\omega_{2}t\\ & =\frac{\alpha_{1}\alpha_{2}^{2}}{4}\left[\cos\left(\omega_{1}+2\omega_{2}\right)t+\cos\omega_{1}t\right]+\frac{\alpha_{1}\alpha_{2}^{2}}{4}\left[\cos\left(2\omega_{2}-\omega_{1}\right)t+\cos\omega_{1}t\right]\\ & +\frac{\alpha_{1}\alpha_{2}\beta }{2}\left[\cos\left(\omega_{1}+\omega_{2}\right)t+\cos\left(\omega_{2}-\omega_{1}\right)t\right]+\alpha_{2}n\left(t\right)\cos\omega_{2}t \end{array}$$

The output signal after low-pass filtering is $S^{\prime }\left(t\right)$ with the expression:

(5) $$S^{\prime }\left(t\right)=\frac{\alpha_{1}\alpha_{2}^{2}}{2}\cos\omega_{1}t$$

Under ideal circumstances, the output signal $S^{\prime }\left(t\right)$ after modulation and demodulation only contains the frequency of the measurement signal, effectively removing the low-frequency noise of the TMR current sensor. To restore the measurement signal $S\left(t\right)$ of the TMR current sensor, it is also necessary to insert an amplifier module with a factor of $2/\alpha_{2}^{2}$ at the position before or after demodulation.

### 3.2. Simulation of TMR Current Sensor Modulating Method

**Figure 5:**
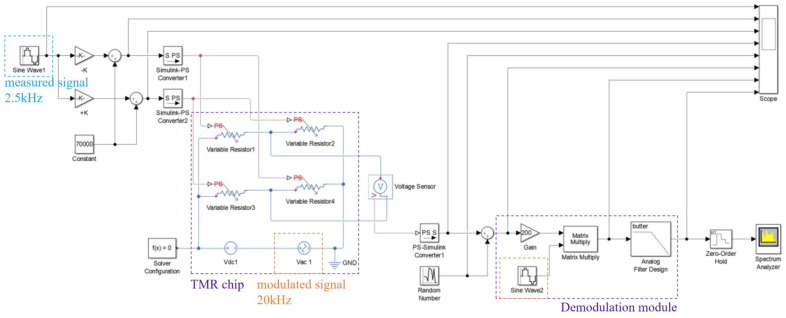
Simulation model of TMR current sensor modulation-demodulation test method.

**Figure 6:**
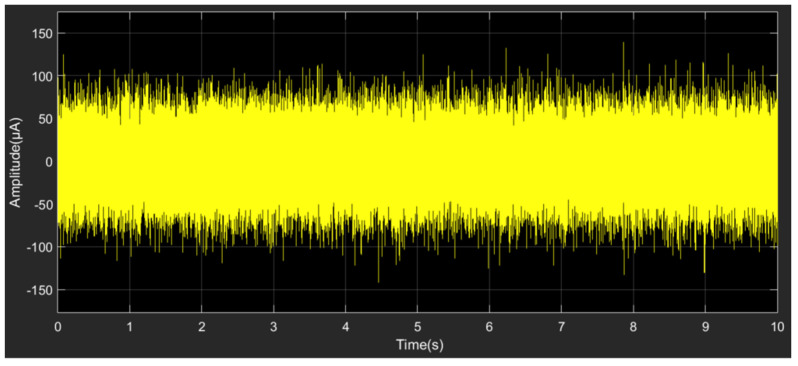
The waveform of noise introduced in the output signal of the TMR current sensor.

The Simulink simulation model of the TMR current sensor modulation and demodulation method is shown in Figure 5. The TMR current sensor consists of four variable resistors whose resistance change is controlled by the input current. The modulation signal in the form of an AC voltage is applied to the sensor supply voltage. For simulating the system and external environmental disturbances during weak current detection, random noise was introduced at the position of the TMR current sensor output, and the noise waveform is shown in Figure 6. The measurement signal with superimposed noise is input to a lock-in amplifier, which includes an amplifier, a phase discriminator, and a filter module.

**Figure 7:**
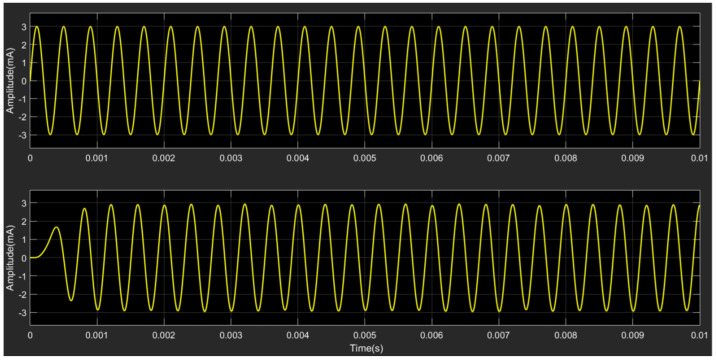
Time domain signal of the standard test current (**upper**) and the demodulated signal (**bottom**).

Figure 7 represents the time domain signal of the standard test current and the model output; the upper waveform is the standard test current, and the bottom waveform is the signal output from the filter after the modulation-demodulation system under noise interference. It can be seen that the system output signal restores the current signal while excluding noise interference. However, due to the inclusion of various parts of the system, the system output signal has a hysteresis.

**Figure 8:**
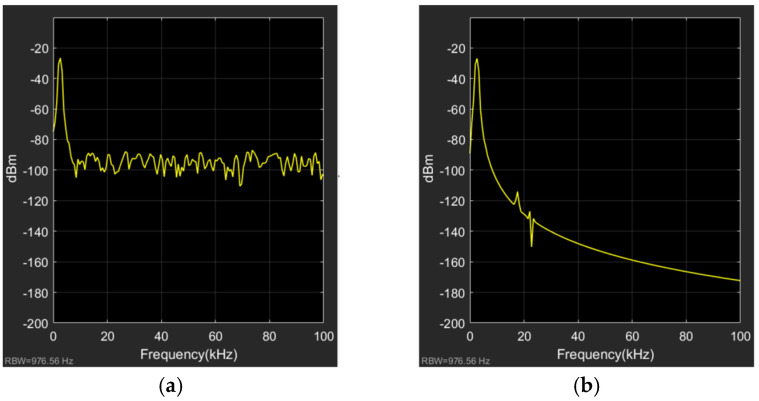
Spectrum of output signal of the system: (**a**) Spectrum of the output signal without modulation-demodulation test method; (**b**) Spectrum of the output signal with modulation-demodulation test method.

To further investigate the noise attenuation effect of the test method proposed in this work, this study established a test system without the modulation-demodulation test method and compared the spectral characteristics of the output signals of two test system models under the same conditions, as shown in Figure 8. The left side shows the spectrum of the TMR current sensor output signal without the proposed test method, while the right side shows the spectrum of the entire system output signal with the inclusion of the proposed test method. Without the modulation-demodulation test method, the spectrum of the TMR current sensor output has a peak at the same frequency as the standard test current and noise at all other frequencies. However, with the inclusion of the proposed test method, except for the peak at the same frequency as the standard test current and at the position near the modulation signal, the noise at all other frequencies is attenuated, which demonstrates the effectiveness of the modulation-demodulation test method.

## 4. Experiment and Discussion

### 4.1. Testing System

**Figure 9:**
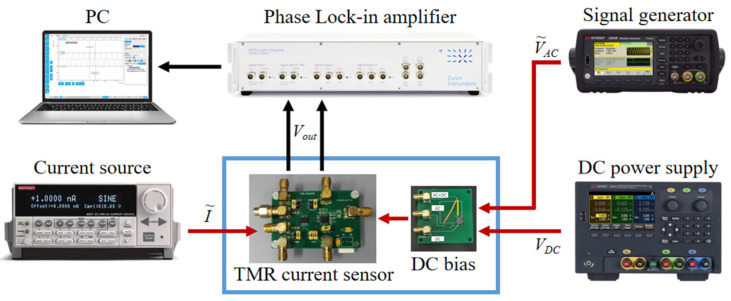
The testing system of the TMR current sensor with the modulation-demodulation test method.

The testing system with the modulation-demodulation test method for the TMR current sensor, constructed based on the simulation model, is shown in Figure 9. The experiment utilizes the MAC005 TMR current sensor chip from MultiDimension Technology Co., Ltd. (Zhangjiagang, China), which has an internal integrated coil as an anti-external magnetic field interference design. The chip’s integrated coil is used to pass the measured current into the chip’s coil pin, and the measured current value is obtained by measuring the differential output voltage across the chip. The DC voltage is provided by the Keysight E36311A DC power supply from Keysight Technologies Inc. (Santa Rosa, CA, USA), while the sinusoidal modulation signal is generated by the Keysight 33500B function signal generator. The DC voltage and AC voltage are loaded onto the TMR current sensor through a DC bias circuit to provide a bias voltage for the internal bridge circuit. To ensure the accuracy of the given measured current, the Keithley 6221 ultra-low current noise AC/DC current source from Tektronix Inc. (Beaverton, OR, USA) is used to provide the TMR current sensor with the AC measured current. The differential output voltage of the TMR current sensor is connected to the input of the Zurich HF2LI 50 MHz lock-in amplifier, which is from Zurich Instruments AG (Zurich, Switzerland). The lock-in amplifier performs demodulation on the differential input signal and sets the low-pass filter parameters to obtain the demodulated measurement signal. The measurement data is transmitted digitally between the instruments and the host PC, and the Plotter, Scope, Spectrum, and other control tools in the host PC software package facilitate real-time analysis of the measurement signal. After exporting the signal, Matlab R2016b is used for data processing. The entire testing system faithfully reproduces the Simulink simulation model of the modulation-demodulation testing method for the TMR current sensor. To study the denoising effect of the modulation-demodulation testing method on the TMR current sensor in practical applications, the entire experiment is conducted in a non-magnetic shielded environment, which includes interference from external stray magnetic fields such as the Earth’s magnetic field and electromagnetic fields.

### 4.2. Results

#### 4.2.1. Sensitivity

**Figure 10:**
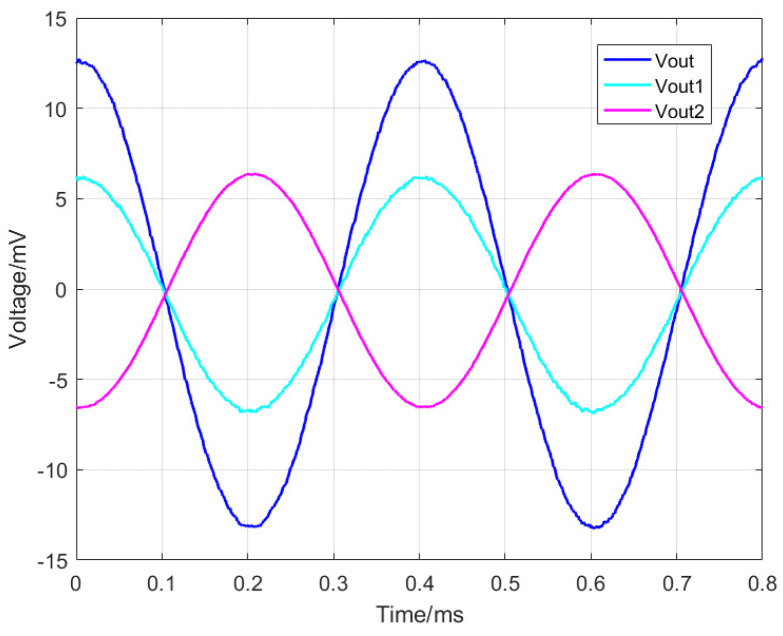
The real-time output waveform of the TMR current sensor when the current is 1 mA.

Magnetoresistive sensors, functioning as resistive sensors, exhibit sensitivity that is dependent on the supplied voltage. The sensitivity of a magnetoresistive current sensor can be defined as the ratio of the sensor’s sensitivity to the supplied voltage. In this experiment, the TMR current sensor is powered by a 5 V supply. The frequency of the measured current is set at 2.5 kHz, with an amplitude ranging from 0 to 10 mA. The phase shift is 0°, and there is no bias. Figure 10 illustrates the real-time output waveform of the TMR current sensor when the current is 1 mA.

**Figure 11:**
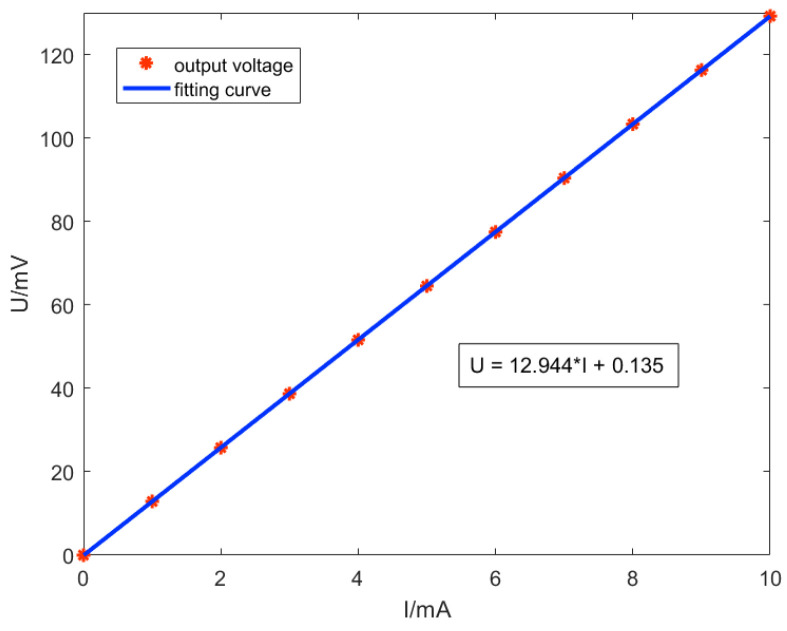
Input-output curves of the TMR current sensors without modulation-demodulation test method.

The response curves of the input current and output voltage of the TMR current sensor are plotted according to the test results from which a linear fitting curve is applied, as shown in Figure 11. It can be observed that when the amplitude of the input AC current is lower than 10 mA, the value of the input current can be deduced from the output voltage of the TMR current sensor using the fitting Equation (6). The sensitivity of the TMR sensor, without the inclusion of a modulation and demodulation method, can be obtained by dividing the slope of the linear fitting curve by the supply voltage, yielding a sensitivity of 2.589 mV/V/mA.

(6) U=12.944×I+0.135

**Figure 12:**
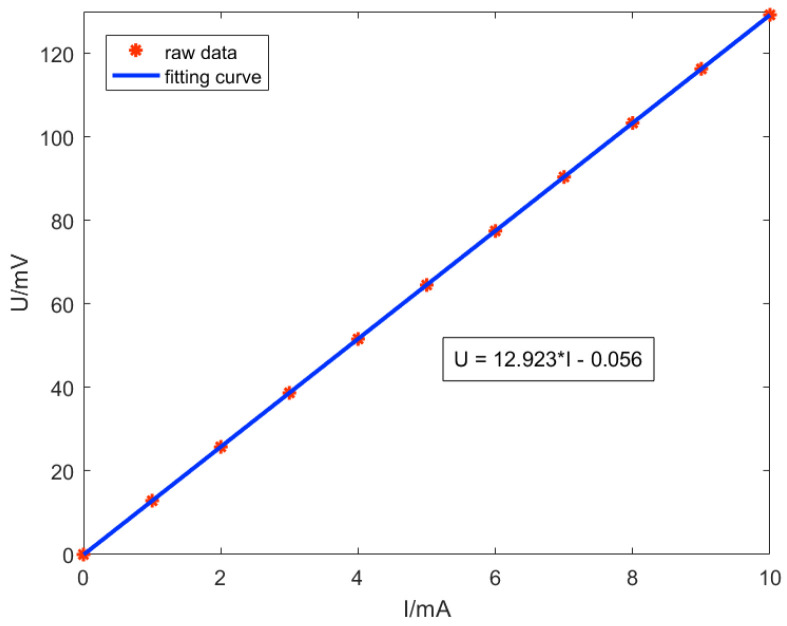
Input-output curves of the TMR current sensors with modulation-demodulation test method.

In the case where the supply voltage and input current are the same as in the previous experiment, a modulation-demodulation test method is introduced with a modulation signal frequency of 20 kHz, an amplitude of 100 mV, and a phase shift of 0°. After demodulation by a lock-in amplifier, the response curves of the input current and output voltage of the TMR current sensor, as well as the linear fitting curve, are shown in Figure 12. The value of the input current can be deduced from the output voltage of the TMR current sensor using the fitting Formula (7). According to the same principle, the sensitivity of the TMR current sensor with modulation and demodulation is calculated to be 2.585 mV/V/mA. When comparing the two cases of modulation and no modulation, it can be seen that this experiment involves frequency modulation, which has almost no impact on the sensitivity of the TMR current sensor.

(7) U=12.923×I−0.056

#### 4.2.2. Nonlinearity

**Figure 13:**
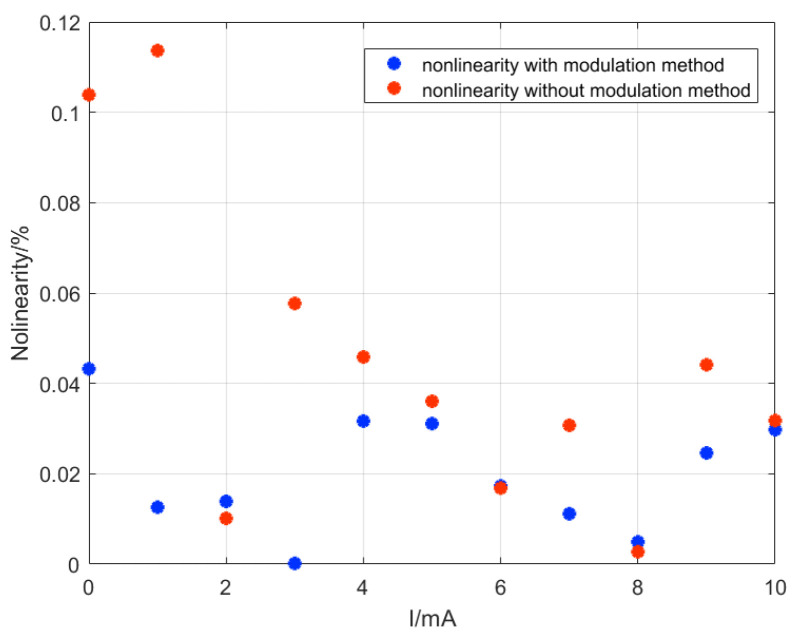
The nonlinearity of the TMR current sensor with and without modulation test method.

Nonlinearity refers to the deviation between the actual input-output curve of the TMR current sensor and the ideal input-output curve. The nonlinearity error of the TMR current sensor in the range of 0–10 mA input current was obtained by subtracting the actual test data from the fitting curve in Figure 11 and Figure 12 and then dividing it by the full-scale range. The nonlinearity of the TMR current sensor with and without modulation is shown in Figure 13. Without modulation, the nonlinearity of the TMR current sensor is 0.12% at maximum. However, with the addition of modulation, the nonlinearity of the TMR current sensor is reduced to 0.045%. This indicates that the inclusion of the modulation-demodulation test method helps to decrease the nonlinear components present in the output voltage of the TMR current sensor, which may originate from system noise and TMR chip drift.

#### 4.2.3. Spectrum

**Figure 14:**
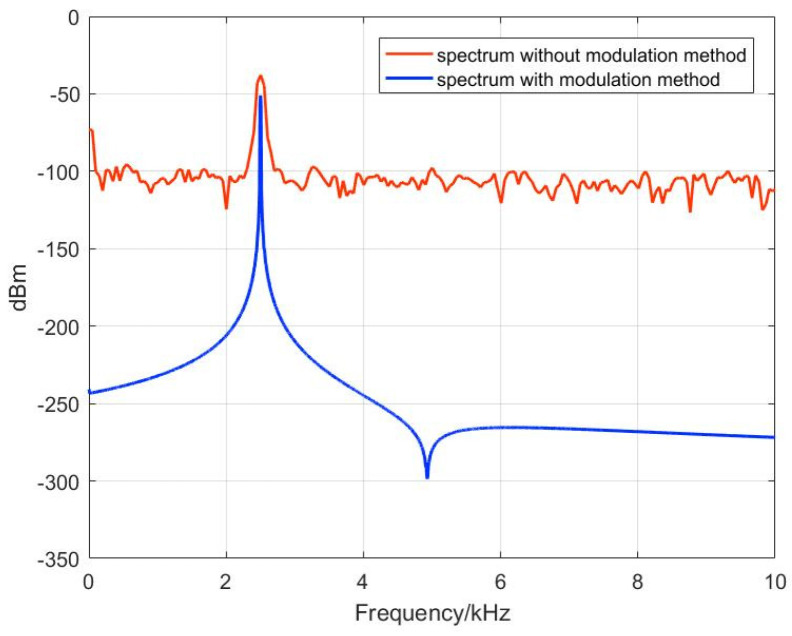
Spectrum of the output signal of the TMR current sensor.

In order to analyze the frequency components of the output signal, it is necessary to transform the time-domain signal into a frequency-domain signal and study it by decomposing it into individual harmonic components using the Fourier transform. The frequency domain structure of the output signal is then analyzed through spectral analysis. The output signal of the TMR current sensor without the modulation method, as well as the output signal under the modulation method, are imported into the PC. The time-domain signals are subjected to a fast Fourier transform (FFT) using Matlab to obtain the frequency-domain signals of the output signals under the two conditions, as shown in Figure 14. The red waveform represents the spectrum of the output signal without the modulation method, while the blue waveform represents the spectrum of the output signal after the addition of the modulation method. As can be seen from the figure, both signals have distinct peaks at the input current frequency of 2.5 kHz. Except for the peak at 2.5 kHz, the spectrum of output signal without modulation method has components with amplitudes in the μV range at various frequencies, as well as significant low-frequency noise. After the introduction of the modulation method, the SNR reaches 114 dB within the bandwidth of the filter. Compared to the SNR of 37 dB in the output signal of the TMR current sensor without the modulation method under the same bandwidth, an increase of 77 dB is achieved. This indicates that the addition of a modulation method can effectively reduce the noise in the output signal of the TMR current sensor. This is because the testing system first modulates the low-frequency noise in the output voltage signal to the high-frequency range and then demodulates and filters out the high-frequency signals, thereby filtering out the low-frequency noise in the output signal.

#### 4.2.4. Resolution

The resolution of the TMR current sensor refers to the minimum input current variation that can be reflected by the output voltage. It is considered distinguishable when the output voltage variation is greater than or equal to half of the theoretically calculated output voltage variation. The theoretically calculated output voltage variation can be obtained by multiplying the sensitivity and the input current variation. In this work, the AC resolution of the TMR current sensor is measured.

**Figure 15:**
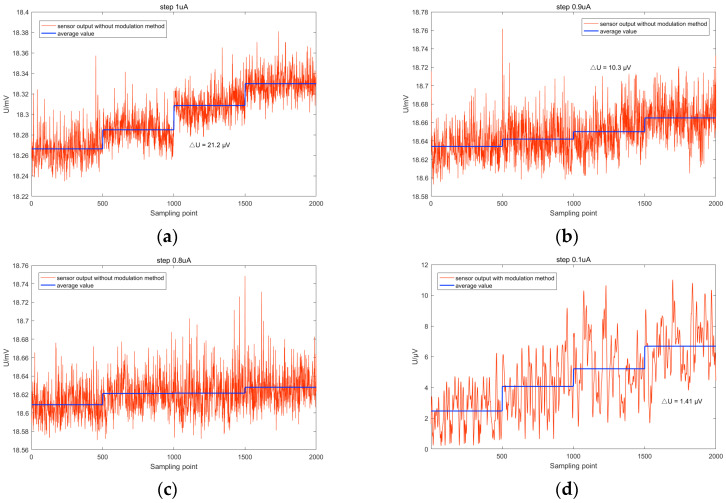
Output voltage of the TMR current sensor for different input current variations: (**a**) variation of 1 μA without modulation-demodulation test method; (**b**) variation of 0.9 μA without modulation-demodulation test method; (**c**) variation of 0.8 μA without modulation-demodulation test method; (**d**) variation of 0.1 μA with modulation-demodulation test method.

In the testing system without the modulation method, the input AC current is initially set to zero and then changed by increments of 1 μA, 0.9 μA, and 0.8 μA, respectively. The corresponding output voltage values are measured. Five hundred output voltage sampling points are collected for each input current, and the average of the sampled voltages is calculated. The variation of the TMR current sensor’s output voltage and the average value of the voltage after averaging are shown in Figure 15. The red waveform in the figure represents the output voltage of the TMR current sensor without the modulation method, while the blue line represents the average value of the sensor’s output voltage for each input current. The average voltage variation ΔU is indicated in the figure. In Figure 15a, when the input current increases by 1 μA, the theoretically calculated output voltage variation can be calculated as 12.944 mV/mA * 1 μA = 12.944 μV, according to the sensitivity obtained in Section 4.2.1. The measured average voltage variation is 21.2 μV, which is greater than half of the theoretically calculated voltage variation, indicating that the TMR current sensor can distinguish a 1 μA input current variation in the testing system without a modulation method. As the input current variation decreases, when the input current increases by 0.9 μA, as shown in Figure 15b, the theoretically calculated output voltage variation is 11.691 μV, and the measured ΔU is 10.3 μV, which is greater than half of the theoretical value, indicating that the resolution of the TMR current sensor is achieved at 0.9 μA. However, when the input current variation is less than 0.9 μA, as shown in Figure 15c, the output voltage of the TMR current sensor for an input current variation of 0.8 μA is measured to have a ΔU smaller than the corresponding theoretical output voltage variation, indicating that the TMR current sensor cannot distinguish input current variations below 0.9 μA. Similarly, it is found that the resolution of the testing system with modulation and demodulation reaches 0.1 μA, as shown in Figure 15d, indicating that the modulation method can effectively improve the AC resolution of the TMR current sensor testing system.

## 5. Conclusions

This study proposes a high-resolution modulation-demodulation testing method to reduce low-frequency noise interference originating from magnetic resistance elements, circuitry, and ambient stray magnetic fields in the measurement of weak currents using TMR current sensors. The modulation-demodulation test method differs from the usual flux modulation for the ultra-sensitive magnetic measurements in that it converts the current to a magnetic field, which results in a variation of the TMR resistance value, and the modulation is realized on the TMR full bridge. Theoretical analysis of the non-contact magnetic resistance bridge structure output within the TMR current sensor was conducted in this study, and a Simulink model for the modulation-demodulation testing method was developed. The accuracy of the model was validated through comprehensive time-domain and frequency-domain simulations. Utilizing the simulation model, a testing system was constructed, with experimental results indicating improvements in the nonlinearity, noise characteristics, and resolution of the TMR current sensor. In an unshielded magnetic environment, implementing the modulation-demodulation testing method reduced the sensor’s nonlinearity from 0.12% to 0.045%, enhanced the signal-to-noise ratio by 77 dB, and increased the AC current resolution from 0.9 μA to 0.1 μA. Furthermore, the modulation approach, involving the addition of an AC modulation signal to the magnetic resistance bridge’s supply voltage, offers simplicity and practicality in testing and can be adapted to reduce low-frequency noise in various resistive elements.

## Data Availability

Experimental data can be obtained by contacting the author via email (yangying212@mails.ucas.ac.cn).
